# Exploring the Social Networks of Women Bereaved by Stillbirth: A Descriptive Qualitative Study

**DOI:** 10.3390/jpm11111056

**Published:** 2021-10-21

**Authors:** Tosin Popoola, Joan Skinner, Martin Woods

**Affiliations:** School of Nursing, Midwifery and Health Practice, Victoria University of Wellington, Wellington 6012, New Zealand; Joan.Skinner@vuw.ac.nz (J.S.); Martin.Woods@vuw.ac.nz (M.W.)

**Keywords:** drawings, Nigeria, perinatal loss, social networks, stillbirth, stillborn

## Abstract

The loss of a baby to stillbirth is a traumatic experience and can lead to secondary losses, such as the loss of social relationships. In Nigeria, stillbirths are a common public health problem. However, limited attention has been given to the social ramifications of stillbirths. This study describes the social networks of women who have experienced a stillbirth and the factors influencing their social networks. Interviews and social network diagrams were used to collect data from 20 women about their social networks before and after stillbirth. Findings suggest that the experience of shame, unmet expectation of support, and a lack of trust led to relationship changes after stillbirth. Most participants met bereavement needs with their existing social networks before stillbirth, but many participants also experienced relationship losses (even among family networks). Information from social network analysis can reveal the risks and strengths inherent in social networks, which can be helpful for the provision of tailored/personalized bereavement care.

## 1. Introduction

The loss of a baby to stillbirth is a devastating and traumatic experience for women [1,2]. However, women do not just lose a baby when they experience stillbirth. When a child is lost to stillbirth, women often lose their emerging social status as an ‘expecting mother’ [3,4], leading to shame and low self-esteem [5] The experience of shame after stillbirth often leads to social withdrawal, loneliness, and relationship deterioration [5,6], which may lead to prolonged or complicated grief [4].

Generally, bereaved people experience changes in their social networks [7,8], and this is no different for mothers of stillborn babies [9,10]. However, in stillbirth bereavement, mothers may feel unsupported and isolated [9,11]. As a result of social withdrawal due to stigma, women’s social networks may become smaller, disconnected, or under-resourced [10,11,12] and their family may emerge as the primary source of support [1,2]. However, the social ramifications of stillbirths extend beyond the family [1,13]. Even if the family was supportive, the bereaved mother would need others outside the family to successfully reintegrate back into society [4]. However, there is limited focus on the social networks of women bereaved by stillbirths.

Social networks, understood here as the people and institutions through which an individual receives and gives social support, is important in stillbirth bereavement. After a stillbirth, women turn to their social networks to seek and receive social support [4]. However, many factors determine whether social support will be exchanged. For example, in spousal bereavement, Morrigan et al. [8] found that unmet expectations of support led to relationship loss and changes. Similarly, Aoun et al. [7] found that the amount, timing, function, and structure of social support influenced bereaved peoples’ perception of the helpfulness or unhelpfulness of their social networks. However, research continues to favor the individual and psychological aspects of stillbirth bereavement over social dimensions of stillbirth loss.

In Nigeria, stillbirths are a significant public health problem. In 2019 alone, 171,428 babies were stillborn in Nigeria [14]. Dated but important studies on perinatal loss in Nigeria suggest that social support protects against depression and anxiety [15,16]. However, social norms have also been reported to prevent new social relationships after stillbirth in Nigeria [17]. For example, research suggests that Nigerian women have little to no opportunities to connect with their social networks after stillbirth due to the absence of rituals and funerals for stillborn babies [17,18]. Despite this, no research has examined the social ramifications of stillbirth loss in Nigeria.

Women’s social networks hold social resources such as thew emotional, financial, and psychological support needed for a healthy grieving. As a result, it is crucial to identify the social networks that women bereaved by stillbirth have access to or do not have access to. This research seeks to describe the social networks of a sample of Nigerian women who have experienced the loss of a baby through stillbirth and the factors influencing their social networks.

## 2. Method

This study utilized a descriptive qualitative design. After obtaining ethical approvals from the Ethical Review Committee of the Saki Baptist Medical Centre and the Human Ethics Committee of the Victoria University of Wellington (#23450), women bereaved by stillbirth were recruited. Twenty women aged 22 to 44 years (mean = 33.5) were recruited through snowballing from Saki, a town predominantly occupied by the Yoruba ethnic group in southwest Nigeria (Table 1). The sociocultural norms and attitudes of the Yoruba people about stillbirth have been described elsewhere (Popoola et al., 2021b). Potential participants who expressed interest in discussing their stillbirth experience were contacted by the first author (TP) who also happens to be a native of Saki town. Since the focus of the research was on the participants’ social networks, eligibility was limited to women whose stillbirth was more than six months but less than three years ago. The eligibility criterion was intended to minimize distress during the early stages of grief and allow a timeframe where mothers could still remember those who played a role in their adjustment to loss.

### 2.1. Data Collection

All women who participated in this study signed written informed consent before data were collected with face-to-face semi-structured interviews and social network diagrams in 2017 (Table 2). Most of the participants (*n* = 19) were interviewed in their homes based on their preferences. Interviews preceded social network diagrams since we wanted to build rapport and ease the participants into talking about a traumatic loss. The drawings were made on a Livescribe A5 notebook with the Livescribe pen since it allowed digital capturing of the diagrams and recording of the interviews.

The instructions that guided the creation of the diagrams were adapted from Pienaar et al.’s [19] communication mapping methodology, an approach that is embedded in communicative ecology models. Broad et al. [20] defined communicative ecologies as the network of connections individuals or groups depend upon to achieve their goals. Thus, from a communicative ecology perspective, the different people who are involved in an ecology, their relationships, and the social institutions and structures that connect them are the focus of analysis [21]. Visually representing social networks through diagrams has been found to facilitate meaningful discussions about social networks and the social context in which giving and receiving social support occurs [22]. In this study, elucidating information through social network drawings was important since women bereaved by stillbirth often perceive that their grief is not real and may be reluctant to discuss their experience publicly [4].

To construct the social network diagram, each participant was asked to draw a picture of herself on the Livescribe A5 notebook. After that, the participant was then asked to add her social networks to the image. Each participant was also asked to join herself with each social network using a maximum of three lines to show the quality of relationships. To identify the changes that might have occurred to the social networks after stillbirth, each participant was asked to look at the diagram and draw another one showing her social networks before stillbirth. After drawing the social network diagrams, the participants were invited to reflect on the changes in their social networks. The participants’ reflections on their drawings were validated against the interview data and any discrepancies were discussed and clarified. The collection of two drawings per participant means 40 diagrams were produced in total. The interviews averaged 45 min.

### 2.2. Analysis

The data collection processes included audio-recordings and digital capture of the diagrams, using a Livescribe A5 notebook/pen. The interviews that were conducted in the Yoruba language were translated to English during transcribing. A back-translation to Yoruba was conducted to ensure translation accuracy, and a second native Yoruba speaker verified this. The back-translation had to consider the challenge presented by the use of metaphors by the participants. Consistent with the nature of the data, we adopted both quantitative and qualitative methods of analysis. Raw images from the participants were used to present their social networks. However, a simple statistical analysis was used to analyze the number of people in the social networks and relationships (such as family). A qualitative analysis (thematic) was performed to explain the factors influencing the participants’ social networks. Starting with data familiarization, the dataset was read repeatedly to identify statements relevant to the participants’ social networks and the factors that determined why a participant turned to someone for help or not. Based on the significant statements identified from the data, the authors jointly developed a codebook and used it to group the significant statements into themes.

### 2.3. Rigor

The criteria for ensuring rigor in qualitative research by Lincoln and Guba (cited in Connelly [23]) was followed in this study. The first author (TP) collected all of the data, and this enhanced the consistency of the data collection procedure. The first author also had the advantage of cultural insights, which enhanced the quality of the data interpretation. The use of culturally appropriate probes also assisted with the achievement of prolonged engagement with the participants. Participants also had opportunities to confirm the accuracy of interview transcripts/summaries and those who did (*n* = 7) made no changes. A reflective journal was kept during the project’s data collection phase, which was used to debrief the co-authors. The findings are grounded in participants’ narratives and all the authors agreed with the final thematic schemata. Pseudonyms SK# was used to present quotations.

## 3. Results

Before stillbirth, the total number of people found in the social network diagrams of the 20 participants was 127. For individual participants, the lowest social network was 2, while the highest social network was 15 (average = 6.35) before stillbirth. After stillbirth, the total number of people found in the social network diagrams of the 20 participants reduced to 99. For individual participants, the lowest social network after stillbirth was 2, while the highest social network was 8 (average = 4.95). The social network analysis revealed six types of social relationships: family, friends, acquaintances, colleagues, neighbors, and healthcare providers. Family networks (consanguineous and affinal kin) accounted for more than half of participants’ social networks before and after stillbirth, while healthcare providers were the least represented (Table 3).

The social network of most of the participants was stable, with 11 participants experiencing no loss or gain in social relationships after stillbirth. This suggests that the majority of the participants met the needs of bereavement with their existing social networks. Eight participants experienced relationship losses after stillbirth, with the most remarkable loss being amongst family relationships, where a total of 15 family members were dropped after stillbirth. Compared to the loss of family networks, the relationship loss in other domains was minimal: acquaintance = 4, friends = 4, colleagues = 4, neighbor = 1. Only one participant gained new social networks (from 2 to 6), with the new additional social networks being acquaintances from the place of worship. Table 3 presents the descriptive statistical analysis of the participants’ social networks before and after stillbirth.

### 3.1. Findings Regarding Women’s Social Networks and the Factors Influencing It

The factors that influenced the participants’ social networks are presented in three categories: the perception of shame, expectation of support, and trust.

#### 3.1.1. The Perception of Shame

“After I put to bed [gave birth], I hid the loss from people because I was ashamed that people might call me a failure…”

(SK1)

“Whenever I saw someone who knew I was pregnant, my heart would skip [nervous] and I always tried to scurry away as if I had done something wrong… I felt like I had some impediments that I needed to be ashamed of”.

(SK5)

As indicated in the above quotes, participants tried to conceal the loss by avoiding social interactions. All participants experienced self-shame, with some likening the shame of stillbirth to other socially stigmatizing events such as incarceration. For example, one participant who concealed the loss by misleading others about her whereabouts used a metaphor about incarceration to discuss how stillbirth had devalued her. The participant said “kini idunu elewon ton’so ago mo owo [what pride does an ex-convict have to raise shoulders in the community]” (SK10).

The participants concealed the loss by engaging in two types of strategies. The first strategy used to avoid shame was the observance of a protracted period of mourning. Most participants exceeded the culturally stipulated period of mourning (40 days) since they were unsure how people would interpret their demeanor and conduct.

“Grieving as a mother whose child passed away is very tricky and challenging. On the one hand, you cannot move on too quickly because people expect a lengthy and genuine portrayal of soberness from you… your conduct should convince people that the loss truly and deeply pained you. On the other hand, you also cannot dwell on it for too long because people expect you to be grateful for your own life… So, grieving a stillborn child is like a performance; the timing of your re-entrance into the society, the way you carry yourself and your countenance must genuinely reflect your sadness but also your gratitude… Performing this role is hard”.

(SK13)

The second type of behavior that the participants used to avoid shame was relocating from their familiar environment. One participant even implied that “*suicide was better than facing shame [iku ya ju esin lo] (SK15)*”. To avoid facing the shame of stillbirth, some participants relocated from their homes for up to six months, thinking that the passage of time would make people forget about the pregnancy. One participant who relocated for over six months said, “*ki oju ma ri ibi, gbogbo ara lo gun e [in order for my eyes not to see shame, I had to flee with all my body]” (SK18)*.

**Figure 1:**
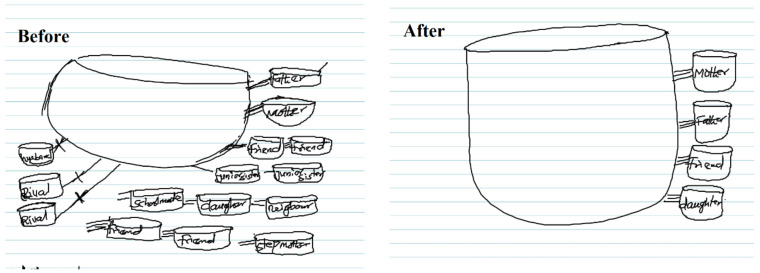
Iku ya ju j’esin lo [death is more preferable to shame] (SK15).

Both strategies employed to avoid shame resulted in relationship loss or inhibited the formation of new social networks. As illustrated in Figure 1, efforts to conceal the loss from others mean the family was more likely to be left in the social network after stillbirth, making the family a critical support network.

Supportive social interactions tend to counteract the negative impact of shame, as one participant described.

“When it happened [stillbirth], it felt like I was alone and I felt like there was nothing good about me anymore. But with the support of my mother, my level of shame started to reduce, and I started to become more comfortable in the presence of others”.

(SK13)

#### 3.1.2. Expectation of Support

After the stillbirth, participants evaluated their relationships based on the perception of received/expected support and the level of interest shown by others in the grief. Using the metaphor below, some participants said they realized that some people in their social networks were fair-weather friends and could not be relied on for support.

“owo epo ni araye n’banila, won ki n’banila t’eje [people want to taste part of the oil (palm oil) in your hands, but not the blood]”.

(SK12)

All relationships were evaluated for their worth and usefulness, but it seemed that women were more critical of family members and close friends in terms of expected support. Unlike other relationships, participants were more likely to describe the support they expected or received from family members as a form of obligation.

“The person [child’s father] who was supposed to help and support me was nowhere to be found. Everything he was supposed to do as a spouse and the father of the child, he did not do”.

(SK12)

“I would have loved my mother-in-law to be there for me, but she ended up disappointing me”.

(SK17)

“I did not feel that those around me understood my grief. Even though people surrounded me, I felt alone… Everyone was saying the same thing and doing the same thing, but I had other needs that nobody cared about”.

(SK14)

**Figure 2:**
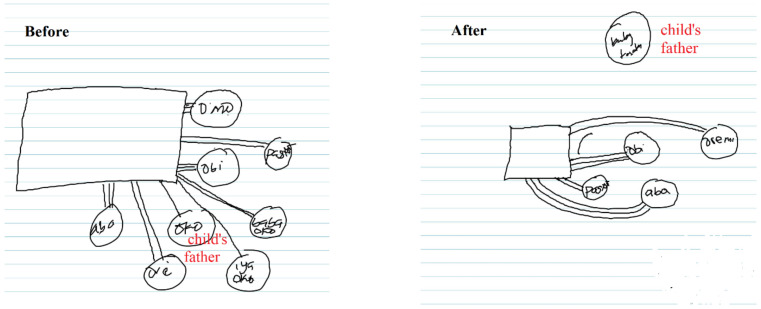
Owo epo ni araye n’banila, won ki n’banila t’eje [people will help you lick your fingers if drenched in palm oil, but not when it is drenched in blood] (SK12).

As seen in Figure 2, family or close social relationships that did not meet the mothers’ expectations tend to be removed from the social network after stillbirth. Single women also tend to have the lowest social network after stillbirth. The highest number of people in single women’s social networks was four (ranged from two to four) and the quality of relationships also tend to be fragile.

**Figure 3:**
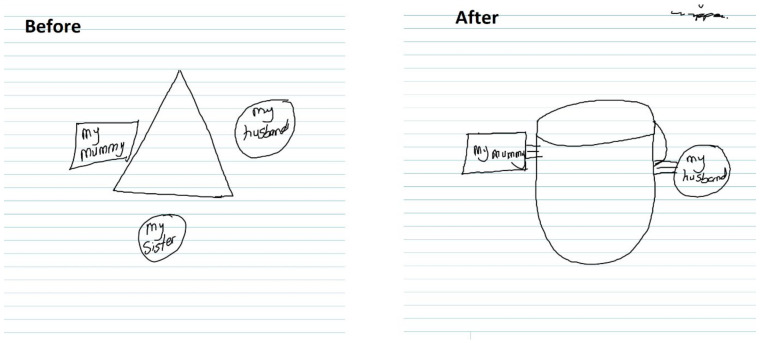
Ti aja ba ni eni lehin, a pa obo [with the support of others, a person can survive any challenges] (SK9).

When social networks met the expectation of support, the quality of the relationship between women and their social networks strengthened. As seen in Figure 3, a participant dropped her sister from her social network after stillbirth. However, the quality of the relationship increased with her mother and husband since they met the participants’ emotional needs.

“My mum was very supportive… she moved closer to me and listened to my views about how I felt about the whole situation. During that time, I needed someone to talk to, not just people who would tell me it will be alright”.

(SK9)

#### 3.1.3. Trust

A lack of trust between participants and their social relations also prevented the formation of new relationships. Certain behaviors such as gossiping, blaming and insensitive comments created distrust and hindered mothers from engaging with others.

“When it happened, I felt like some people thought I deserved to lose the child because I had a home birth. Whenever I walk past, they gossip about me and I sometimes overhear them saying, ‘look at the woman who did not value the life of a child’… I came out of the experience as a very introverted and paranoid person”.

(SK17)

“After the incident, the doctors and the nurses did not even give me the chance to gather myself together before they started asking how we were going to pay the hospital bills… they did not show any human feeling and I did not trust them to look after me”.

(SK10)

While a lack of trust prevented the formation of social relationships, participants bonded with those who were deemed trustworthy in their social network. The majority of the participants received the most support from their spouses and found them trustworthy.

“From my experience, I think the greatest gift you can receive when you lose a child is a true friend that you can trust with your pains… My husband was the one who really stood by me. He didn’t go to work until two weeks after the incident…”.

(SK2)

## 4. Discussion

The findings of this study confirm earlier studies that have reported that bereavement leads to relationship changes [7,8,10]. The factors that influenced relationship changes in this study: shame [5,13], the unmet expectation of support [2] and mistrust [2,10] have been confirmed separately by other studies. Of the three factors that influenced the participants’ social networks, shame/stigma has received the most attention in stillbirth research. In response to perceived/anticipated shame, women may try to conceal the loss [5,13], leading to relationship changes [2,10]. Consistent with earlier studies [5,6], we found that our participants employed strategies such as relocating or observing prolonged mourning to avoid shame, but this resulted in relationship loss or stagnancy. While concealment strategies might provide temporary salve for mothers, Pollock et al. [5] note that such strategies might heighten the risk of internalized grief and guilt, which may worsen bereavement outcomes in the long term.

In times of loss and crisis, people commonly turn to their social network for support [4]. However, being embedded within a network of relationships does not guarantee support for bereaved people [7]. Factors that determine whether support will be perceived as helpful or unhelpful have been explored from the size or quality of social networks but with conflicting results. For example, among people who have experienced traumatic events, Platt et al. [24] found that heterogeneous social network, not perception of strong social support, was more protective against post-traumatic stress disorder. In contrast, Ferlander et al. [25] found that a homogenous social network comprising of immediate family was more protective against depression for Russian women. Limited studies on bereavement, including stillbirths, suggest that type, size, and quality of social networks are essential for bereavement outcomes [7,26].

The needs of bereaved people are often complex and multifaceted [7,8]. Common needs during stillbirth bereavement, such as rebuilding identity, planning funerals, resolving psychological trauma, planning future reproductive health, and meeting financial needs, may be best met by a combination of intrapersonal and interpersonal social networks [1,27]. In this study, the participants’ social networks did not include stillbirth support groups and there was an under-representation of health personnel. In addition, the participants who were single also tend to have smaller social networks and this echoes earlier studies that have reported that unmarried women have limited support after stillbirth [26,28]. This finding highlights that single women and those whose social networks are devoid of stillbirth support groups and health professionals may have limited support and feel socially isolated.

Similar to earlier studies [2,10], our findings also suggest that women rely more on family networks than any other types of relationships. In stillbirth bereavement, women may have no other choice but to rely on their family and existing social networks due to stigma and lack of social recognition of stillbirth loss. However, while the family may not be able to meet all the bereavement needs, Cacciatore et al. [26] found that family support is the only type of support that predicted anxiety and depression. Our findings replicate previous studies that family support is not a guarantee in stillbirth bereavement [2,6]. Previously, it has been reported separately that incongruent grieving styles, migration and insensitive comments from family members lead to family breakdown after stillbirth [2,6,29]. Our study adds that moral obligations within family relationships can play dual roles in bereavement; it can guarantee support for mothers, but also increase the likelihood of family breakdown. Family crises pose a severe threat to mothers’ wellbeing and bereavement outcomes, especially in low and middle-income countries where social support groups, trained counsellors, and psychiatrists may not always be available [27]. Therefore, health personnel must recognize that the family network may be under pressure to meet bereavement needs, especially when external support is limited.

In this study, the mean social network of mothers after stillbirth was 4.95 (range 2–8). This is smaller compared to women in general, where an average number of 10 people has been reported among women’s social networks [30,31,32]. The paucity of evidence on the social network of mothers of stillborn babies means we cannot compare the size of social networks that we found with other studies. However, it is important for those caring for women bereaved by stillbirth to know whether they should focus their interventions on social networks’ size/quality, or structure/contents. Future studies are needed to increase our understandings of aspects of social networks that influence bereavement outcomes.

## 5. Strength and Limitations

This study employed a creative and novel method to facilitate dialogue about a tabooed and challenging subject. The use of drawings permitted metaphors, which unlocked the barriers posed by language and social norms around the issue. The use of dual media of interviews and drawings allowed the participants to express feelings that may have been difficult in words. The limitations in this study are related to the methodological decisions taken. First, the participants’ social network may be more extensive than we found if this study was conducted immediately after stillbirth. Second, the network size that we found may differ if the participants were asked to list or estimate their social networks instead of drawings (see Morrigan et al. [8], for instance). Third, time since loss might have resulted in the under-estimation of the social networks, but we did not notice any significant difference between participants based on time since the loss. Fourth, real-life relationships are intertwined and conceptually distinguishing them by the nature of relationships might overlook some nuances in the way people experience their social relationships. Fifth, due to the small sample size and the fact that all the participants belonged to the same Yoruba ethnic group, the transferability/generalizability of the findings to other contexts need to be assessed carefully. However, while the social networks that we found in this study might not represent all possible ties and relationships, it presents the social network that the participants identify as significant for themselves after stillbirth.

## 6. Conclusions and Practice Recommendations

The loss of social networks and the inability to form new social networks after stillbirth suggests that mothers of stillborn babies may feel unsupported, isolated and miss out on critical bereavement support. Our study adds to the emerging literature on social networks of bereaved people [7,8] by suggesting that bereaved mothers’ social networks can be influenced by shame, mistrust and unmet expectation of support.

The use of drawings to capture the social networks of mothers proved to be useful in analyzing the social relationships and the risk factors for social isolation. Since the experience of grief is unique and individual, the social network technique used in this study can help health personnel to provide personalized/person-centered bereavement care. When diagrams are used to elucidate the social relationships of women bereaved by stillbirth, health personnel may find that mothers’ social networks are conflictive, broken or fragmented and may be tempted to want to fix such broken relationships. However, Sanicola [33] argued that the goal of care is not to remedy broken ties but to facilitate collective caregiving. This means that healthcare providers can achieve better bereavement outcomes if they locate bereavement care within the context of supportive and reliable relationships.

Despite the loss of family networks, the family was still a reliable source of support for the participants. However, the vulnerabilities of family relationships to breakdown cannot be overlooked in clinical care. Family breakdown can negatively impact the bereavement outcome, especially in Nigeria, where there is no external support for mothers after stillbirth [11,17]. Due to the risk that stillbirth presents to women and their families, healthcare providers need to approach stillbirth bereavement from a family-centered perspective. Healthcare providers can help mothers and their families discuss expectations and link the family with appropriate and supportive resources.

## Figures and Tables

**Table 1:** Demographic characteristics of the study participants.

	N = 20
**Age in years**	
Mean (range)	33.5 (22–44)
**Educational level**	
No education	7
Primary/secondary/Tertiary	3/2/8
**Marital status**	
Married or cohabiting	15
Single	4
Widowed	1
**Time of death of baby**	
Intrapartum (during childbirth)	17
Antepartum (before childbirth)	3
**Place of birth**	
Healthcare facilities	19
Homebirth	1
**Gestational age at the time of loss**	
37 weeks and above	16
30–36 weeks	4
**Gravidity (number of previous pregnancies before stillbirth)**	
Primigravida	8
Multigravida	12

**Table 2:** Interviews and diagrams’ instructions.

Semi-Structured Interview Guide	Social Network Diagrams
1. What was your relationship with friends, neighbors, colleagues, family and others like after loss?	1. I would like you to draw an image of yourself.
2. Could you tell me how the loss of your child impacted on your relationship with others?	2. I would like you to add images of people (friends, neighbors, partner, extended families, colleagues) that come to mind when you think of the loss of your child.
3. What are those things that you can say assisted you to deal with your loss and where did you receive those from?	3. I would like you to add images of any social/environmental systems (hospital, church, mosque, midwife/nurse/doctor, school, work, child support agency) that played either positive or negative roles in your experience of loss.
4. How well would you say you were supported by your relatives, friends, colleagues and others after the loss of your baby?	4. Please join yourself to the people you have added to your drawing with a maximum of three lines and a minimum of one line, depending on how you perceive the person’s support.

**Table 3:** Description of the participants’ social networks.

	Before Stillbirth N = 20 Diagrams	After Stillbirth N = 20 Diagrams
**Social networks**		
Total network size	127	99
Range	2–15	2–8
Mean	6.35	4.95
**Composition of social networks**		
Family	65	50
Friends	34	30
Acquaintance	16	12
Colleagues	7	3
Neighbors	4	3
Healthcare providers	1	1
**Gender composition of social networks**		
Female	98	69
Male	29	30

## Data Availability

The de-identified transcripts of interviews and diagrams are still held by the primary author and can be provided upon reasonable request and sound methodological justification.
